# Body silhouette, menstrual function at adolescence and breast cancer risk in the E3N cohort study

**DOI:** 10.1038/sj.bjc.6602620

**Published:** 2005-05-24

**Authors:** B Tehard, R Kaaks, F Clavel-Chapelon

**Affiliations:** 1 Equipe Inserm ‘Nutrition, Hormones et Cancer’, Institut Gustave Roussy, 39 rue Camille Desmoulins, 94805 Villejuif Cedex, France; 2 International Agency for Cancer Research, Lyon, France

**Keywords:** breast neoplasms, cohort study, risk factors, body shape, childhood, overweight

## Abstract

We analysed the relation between adult breast cancer risk and adiposity in ages 8–25, and among 90 509 women included in the E3N cohort study, and investigated the potential modification effect of certain factors. Participants completed a questionnaire that included a set of eight silhouettes corresponding to body shape at different ages. During the follow-up (mean=11.4 years), 3491 breast cancer cases were identified. Negative trends in risk of breast cancer with increasing body silhouettes at age 8 and at menarche were observed, irrespective of menopausal status, with relative risks of 0.73 (0.53–0.99) and 0.82 (0.66–1.02) for women who reported a silhouette equal or greater than the fifth silhouette at age 8 and at menarche, respectively. We observed no clear effect modification by age at menarche, delay between age at menarche, regular cycling, regularity of cycles in adult life or body mass index at baseline.

Adult adiposity is positively associated with postmenopausal breast cancer risk and may be negatively associated with premenopausal breast cancer risk (Choi *et al*, 1978; La Vecchia *et al*, 1987; WCRF, 1997; COMA, 1998; van den Brandt *et al*, 2000; Friedenreich, 2001; IARC, 2002; Okasha *et al*, 2003). It still remains unclear, however, whether or not it is mostly excess weight during puberty and adolescence that explains the inverse relation of breast cancer risk with premenopausal overweight. In a number of studies (Friedenreich, 2001; IARC, 2002; Alghren *et al*, 2004), premenopausal breast cancer risk was inversely related to recalled weight and body mass index (BMI) around the age of 18, whereas the relation to weight gain since that age remains unclear, one literature review indicating a direct relation (Friedenreich, 2001) and another indicating an inverse relation (IARC, 2002). Among studies on the relation between overweight and breast cancer, seven case–control studies (Hislop and Coldman, 1986; Pryor *et al*, 1989; Brinton and Swanson 1992; Franceschi *et al*, 1996; Hu *et al*, 1997; Magnusson *et al*, 1998; Coates *et al*, 1999), one historical cohort study (Le Marchand *et al*, 1988) and six prospective cohorts (Hilakivi-Clarke *et al*, 2001; Swerdlow *et al*, 2002; Alghren *et al*, 2004; De Stavola *et al*, 2004; Weiderpass *et al*, 2004; Baer *et al*, 2005) have examined the relation between breast cancer and adiposity in childhood. Only two studies (De Stavola *et al*, 2004; Baer *et al*, 2005) investigated the interaction between adiposity, between 2 and 4 years of age and age at menarche in their relation to breast cancer. Studying the events occurring during the period in life of the mammary gland growth may give new insights into the aetiology of the disease.

We have examined the relation between breast cancer and body shape at adolescence, using the data from the E3N study, a large prospective cohort of French women, followed up from 1990 until 2002 (Clavel-Chapelon, 2002).

## MATERIAL AND METHODS

**Figure 1:**
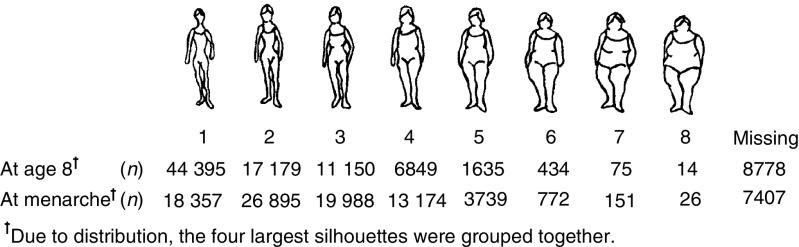
Body silhouettes used in baseline questionnaire (as first proposed by Sörensen *et al*, 1983), with frequency distribution of women's responses.

The E3N cohort consists of 98 995 women living in France, covered by a national health insurance scheme primarily covering school teachers. Participants were aged 40–65 years when they were first recruited into the cohort, between June 1990 and November 1991, by responding to the first in a series of mailed questionnaires during their follow-up. The baseline questionnaire contained questions on established risk factors of breast cancer including aspects of reproductive life, menopause, history of benign breast disease, breast cancer in first-degree relatives and anthropometric measures. Women were also asked to report which of a series of Sørensen's silhouettes (Sørensen *et al*, 1983; www.e3n.net) best described their body shape around the age of 8, at menarche and at age 20–25 (Figure 1); more than 86% of women completed such questions (Figure 1).

Follow-up questionnaires were sent out approximately every 2 years thereafter. All questionnaires asked whether breast cancer had been diagnosed, requesting the addresses of their physicians and permission to contact them. Deaths in the cohort were detected from reports by family members and by searching the insurance company (MGEN) file, which contains information on vital status. Information on cause of death was obtained from the National Service on Causes of Deaths (http://sc8.vesinet.inserm.fr:1080/accueil_fr.html). Information on nonrespondents was obtained from the MGEN file on reimbursement of hospital fees. The third follow-up questionnaire sent out contained a dietary questionnaire. Participants of the E3N cohort who responded to the dietary questionnaire (*n*=74 524) were included in the European Prospective Investigation into Cancer and Nutrition (EPIC) (Riboli and Kaaks, 1997).

Menopause, if applicable, was recorded in each follow-up questionnaire. To promote accuracy of the constructed menopause variables, all answers on date and type of menopause (natural or the result of bilateral oophorectomy, chemotherapy, radiotherapy or other treatment), date of last menstruation, date of start of menopausal symptoms and date of hysterectomy, if appropriate, were reviewed. Postmenopause was defined as the cessation of periods for natural reasons or not.

Follow-up time was between the return of the baseline questionnaire in 1990 and July 2002, when the seventh questionnaire was sent out. Person-years were accrued up to the date of breast cancer diagnosis, death, last questionnaire returned or July 2002 (for replies to the questionnaire received after July 2002), whichever occurred first. Women with a null follow-up were excluded (*n*=2601) from the analyses, as those who declared a prevalent cancer other than a basal cell carcinoma and an incident cancer other than a breast cancer (*n*=5447). Also excluded were women with an incident ductal carninoma *in situ* (*n*=405). Finally, 90 509 women were included in the analyses; mean follow-up was 11.4 years (s.d.=2.4 years).

Owing to the high percentage of pathology reports finally obtained (covering 94.9% of the breast cancers reported up to the sixth questionnaires) and because of the high rate of histologic confirmation (97.8% of these), we decided to consider in the present analysis reported breast cancer cases not yet confirmed (*n*=527). Overall, the present analysis is based on 3491 breast cancer cases, 930 diagnosed before the menopause and 2561 women after their menopause.

Statistical analyses were made using Cox's proportional-hazard models, with subjects' age as the time scale. As menopausal status changed during follow-up for 45 573 women, it was included in Cox's models as a time-dependent variable in analyses that were not stratified by menopausal status. The other adjustment variables taken into account were: adult height divided into quartiles (cut points: 158, 162 and 165 cm), history of breast cancer in first-degree relatives (yes/no), age at menarche (cut points: 12, 13 and 14), age at first full-term pregnancy (FFTP) (cut points: 23, 26 and 30), parity (0, 1–3 and4+), history of benign breast disease (yes/no), alcohol consumption (g of alcohol per week), number of years at school (cut points: 0, 5, 9, 13 and 15), marital status (if ever married or not), oral contraceptive use (yes/no) and physical activity (quartiles of weekly energy expenditure for recreational and household activities cited in the first questionnaire). Additional adjustments were made for BMI at recruitment, the interval between menarche and the establishing of regular menstrual cycles. The four largest silhouettes were grouped together, according to the distribution.

## RESULTS

Evidence for the following risk factors of breast cancer in the E3N population were found (Table 1): early age at menarche, late age at first birth, high height, low physical activity, high educational level, familial history of breast cancer and personal history of benign breast disease. Breast cancer cases on average also reported a smaller silhouette than noncases, both at 8 years of age (*P*<0.0001) and at menarche (*P*<0.0001). Concerning silhouette at age 20–25, no difference was observed between cases and noncases. Body silhouette at age 8 and at menarche were correlated (Spearman's correlation coefficient=0.63, *P*-value <0.0001). Among women who reported a silhouette at both ages (*n*=80 956), 45.6% chose the same silhouette and 83.5% chose one at menarche that was equal or adjacent to that at age 8. Body silhouette at age 20–25 was found less correlated with that at age 8 (Spearman's correlation coefficient=0.62, *P*-value <0.0001) than with that at menarche (Spearman's correlation coefficient=0.44, *P*-value <0.0001).

Patterns of risks with silhouette at age 8 and at menarche were similar among pre- and postmenopausal women (Table 2), with significant negative trends in risk. In the whole group of pre- and postmenopausal women, we observed a linear trend of decrease in risk of breast cancer with increasing silhouette, both at age 8 (*P*<0.001) and at menarche (*P*<0.0005), with relative risks (RR) equal to 0.86 (95% confidence interval (CI): 0.75–0.99) and 0.89 (0.80–0.99), for women who chose the fourth silhouette at age 8 and at menarche, respectively, and equal to 0.80 (0.63–1.02) and 0.90 (0.76–1.06) for women who chose a silhouette equal or greater than the fifth silhouette at age 8 and at menarche, respectively, as compared to women who chose the first silhouette (data not shown). Considering silhouette at menarche, we observed a weak increase in risk among women who chose the second silhouette (RR equal to 1.11 (1.02–1.21)), as compared with the first. The linear decreases in risk observed with silhouettes at age 8 and at menarche were slightly more accentuated after additional adjustment for adult BMI, adult regularity of menstrual cycles and for interval between menarche and regular cycling, with RRs equal to 0.73 (0.53–0.99) and 0.82 (0.66–1.02) for women who chose a silhouette equal or greater than the fifth silhouette at age 8 and at menarche, respectively, as compared to women choosing the first (data not shown). No significant linear relation was found between silhouette at age 20–25 and breast cancer risk, irrespective of menopausal status. However, we observed an increased risk of premenopausal breast cancer among women who chose the second silhouette at this age, with RRs of 1.35 (1.07–1.70), as compared to those choosing the first silhouette (Table 2). Later adjustments for adult BMI, adult regularity of menstrual cycles and for interval between age at menarche and age at regular cycling did not materially change the results.

Logistic regression models (Table 3) to determine if menstrual-related variables and BMI in adulthood were associated with adiposity during childhood. Compared to women with a silhouette of 4 or less at menarche, those with a silhouette greater than or equal to 5 tended to be younger at menarche (*P*_trend_<0.0001, odds ratio (OR)=0.57 (0.52–0.62) for age at menarche ⩾14 *vs* <12), had a longer interval between menarche and regular cycling (*P*_trend_<0.005, OR=1.24 (1.15–1.34) for an interval >2 years *vs* 0), had more often irregular menstrual cycles in adult life (OR=1.16 (1.07–1.27)) and were more adipose at baseline (*P*_trend_<0.0001, OR=1.70 (1.46–1.97) for women in the fourth *vs* women in the first quartile). Having a large silhouette at adolescence was not related to age at regular cycling, except when the latter occurred after age 15 (OR=1.18 (1.10–1.28)). Similar results were observed for silhouette at age 8.

Subgroup analyses (Table 4) indicated similar patterns of risks with silhouette at age 8 irrespective of age at menarche, delay between age at menarche, regular cycling, regularity of cycles in adult life or BMI at inclusion. Similar conclusions were found for adiposity at menarche. No test for heterogeneity between trends in risk by subgroups reached significance.

## DISCUSSION

Our results support the hypothesis of a protective effect of adiposity at young ages on breast cancer risk, irrespective of menopausal status, although the magnitude of the effect was not strong and although the CIs around the RRs for obese girls often included unity. Since adjustment for menstrual and anthropometric characteristics in adulthood did not attenuate our estimates, our results suggest that adiposity during adolescence may have an independent protective effect against breast cancer. No clear association was found between silhouette at age 20–25 and risk.

Few studies have focused on the relation between high weight at (or around) menarche and risk (Le Marchand *et al*, 1988; Franceschi *et al*, 1996; Hu *et al*, 1997; Magnusson *et al*, 1998; Coates *et al*, 1999; Alghren *et al*, 2004; De Stavola *et al*, 2004; Weiderpass *et al*, 2004). Most of these studies showed results quite similar to ours, with a reduction especially of premenopausal risk among women who had a high BMI during childhood (Le Marchand *et al*, 1988; Coates *et al*, 1999; Weiderpass *et al*, 2004). A case–control study nested within a historical cohort in Hawaii (Le Marchand *et al*, 1988) showed a significant negative association of premenopausal risk with high body mass at age 10–14. A lower risk was found for women who considered themselves heavier than average at ages 12–13 and 15–16 (Coates *et al*, 1999). The use of Sørensen's body silhouettes for adiposity at age 7, showed a significant and strong negative association of increasing body silhouette at that age with postmenopausal breast cancer risk, with a three-fold RR for those who had chosen the leanest shape as their age 7 silhouettes, as compared to the largest (Magnusson *et al*, 1998). Three prospective cohort studies have found a significant decrease in risk with childhood adiposity: a Scandinavian cohort study (Weiderpass *et al*, 2004) showed a decreased risk of premenopausal breast cancer among women who were the heaviest girls at age 7 (RR=0.69 (0.51–0.93)), when compared to the thinnest; a high BMI at age 14 was associated with a RR of 0.84 (0.75–0.94) in a Danish cohort (Alghren *et al*, 2004), while another study found a reduced risk only with a high BMI at ages 2–4 (De Stavola *et al*, 2004). Two others studies of anthropometric data at age 12 had no significant results (Franceschi *et al*, 1996; Hu *et al*, 1997). Some studies have indicated that weight may be a risk modifier even earlier in life (De Stavola *et al*, 2004). Unfortunately, birth weight and weight, or adiposity, before age 8 were not available in our study. Overall a decreased risk of premenopausal breast cancer has been found with increasing adiposity around 20 years of age, while such relation was less clear among postmenopausal women (IARC, 2002; Weiderpass *et al*, 2004). Overall our results were globally nonsignificant whatever the menopausal status.

Excess adiposity can alter the production of hormones, notably by increasing the frequency of anovulatory cycles (Stoll, 1997, 1998), which leads to a decrease in progesterone levels. In our data, the percentage of women for whom menstrual cycles became regular more than 1 year after menarche – which may indicate the occurrence of anovulatory cycles – increased from 29 to 42% with increasing body silhouette at menarche. Several studies associated irregular menstrual cycles during life course to a lower risk of breast cancer (Layde *et al*, 1989; Parazzini *et al*, 1993; Den Tonkelaar and de Waard, 1996). Our observations and the fact that the relationships observed were not modified by menstrual and anthropometric characteristics in adulthood suggest that the inverse relation of excess weight during childhood with risk later in life, before or after menopause, may be explained by hormonal mechanisms in the peripubertal period, when mammary tissue develops.

As body silhouettes at age 8 and at menarche are highly correlated, it is difficult to deduce if the protective effect in our study is due to body fatness at age 8, to body fatness at menarche or to both. To try to disentangle these two efforts, we examined the associations between risk and overweight at age 8 among women with a silhouette lesser or equal to 3 at menarche, and also overweight at menarche among those with a silhouette lesser or equal to 3 at age 8. Both analyses showed similar decreases in risk with increasing body silhouette, although the decrease remained significant only with increasing silhouette at menarche (*P* for trend <0.01), perhaps indicating that body fatness at menarche is more relevant to the decreased risk observed in this study.

As E3N participants have high levels of education and health consciousness, our data can be considered reliable with very few missing replies (around 5% for anthropometric variables). As a prospective cohort, recall bias is prevented. The frequent updating of our data (a questionnaire sent out every 2 years) allowed us to determine accurately the menopausal status of the women and to take account of this evolution in our analyses. Although we included breast cancer cases that were not histologically confirmed, the great concordance between self-declaration of cancer and pathology reports allowed us to strengthen the statistical power of our study, and any misclassifications would only bias our estimates towards unity.

However, the fact that the E3N cohort was not population based may reduce the variability of many characteristics and consequently bias our estimates towards unity. This study is based on long-term memory of adiposity between childhood and young adulthood, which may generate important error measurements because many people cannot precisely evaluate their weight and height in childhood, either because of difficulty of accurate recall measurements or because they were not informed of their weight when young. Nevertheless, several previous studies have shown a reasonably high reliability of recalled body weight data, even after long intervals (Stevens *et al*, 1990; Casey *et al*, 1991; De Fine Olivarius and Andreasen, 1997). Moreover, the use of Sørensen's body silhouettes greatly facilitates the distant recall of body shape and adiposity, during childhood. The use of silhouettes was validated to estimate 33 years prior body shape on 448 women, and showed that recalled silhouettes were overestimated by the thinnest girls and underestimated by normal/heavier girls (Must *et al*, 2002). However, such misclassifications would only bias our estimates towards unity. Silhouettes may therefore offer an easier and more accurate estimate of categories of past obesity.

In summary, our results support the hypothesis that obesity during childhood or adolescence reduces breast cancer risk, and that this reduction is not fully explained by menstrual characteristics after menarche nor by adult BMI, but these results need confirmation; further research is also required to analyse the hormonal characteristics of overweight adolescents and to assess whether hormonal modifications related to overweight at adolescence persist in adult life.

## Figures and Tables

**Table 1:** Baseline (1990) characteristics^a^ of breast cancer cases and noncases, E3N study

	**Cases (*n*=3491)**	**Noncases (*n*=87 018)**	***P*-value^b^**
Age at inclusion (years)	50.0 (6.4)	49.2 (6.7)	<0.0001
Age at menarche (years)	12.7 (1.4)	12.8 (1.4)	<0.01
Age at first birth (years)	25.4 (4.3)	24.8 (4.1)	<0.0001
Number of full-term pregnancies	1.9 (1.2)	2.0 (1.2)	<0.0001
Years of education	13.9 (2.1)	13.4 (2.4)	<0.05
Alcohol consumption (g day^−1^)	10.6 (13.8)	10.7 (14.0)	NS
Married	77.5%	77.7%	NS
Oral contraceptive users	39.3%	41.4%	<0.05
Benign breast disease cases	30.2%	22.7%	<0.0001
First-degree relative breast cancer	21.3%	12.4%	<0.0001
Height (cm)	162.0 (5.8)	161.7 (5.7)	<0.005
Physical activity^c^ (METs)	45.4 (26.9)	47.1 (28.2)	<0.005

*Silhouettes*			
At 8 years old	1.7 (1.1)	1.9 (1.1)	<0.0001
At menarche	2.4 (1.2)	2.5 (1.2)	<0.0001
At age 20–25	2.5 (0.9)	2.6 (1.0)	NS

NS=nonsignificant.

a Mean (s.d.) or percentages.

b Calculations were made by *t*-tests and *χ*^2^ tests.

c Weekly energy expenditure for recreational and household activities cited in the first questionnaire.

**Table 2:** RRs of breast cancer by body silhouette at different ages. E3N study, 1990–2000

**Variables**	**Cases**	**Person-years**	**Crude RRs**	**Multivariate RRs^a^**	**Multivariate RRs^b^**
*Premenopausal women*					
Silhouette at age 8					
1	490	155 593	1.00 (reference)	1.00 (reference)	1.00 (reference)
2	173	68 759	0.85 (0.71–1.01)	0.84 (0.71–1.00)	0.88 (0.71–1.09)
3	124	44 808	0.93 (0.76–1.13)	0.91 (0.75–1.11)	0.95 (0.74–1.21)
4	60	26 709	0.75 (0.57–0.98)	0.73 (0.56–0.96)	0.69 (0.49–0.97)
⩾5	21	7813	0.84 (0.54–1.32)	0.84 (0.54–1.32)	0.82 (0.46–1.47)
*P* for trend^*^			<0.01	<0.01	<0.05

Silhouette around menarche					
1	171	60 763	1.00 (reference)	1.00 (reference)	1.00 (reference)
2	315	99 207	1.23 (1.03–1.47)	1.23 (1.03–1.47)	1.20 (0.97–1.50)
3	224	78 310	1.11 (0.92–1.34)	1.13 (0.93–1.37)	1.03 (0.82–1.31)
4	134	52 716	0.98 (0.79–1.21)	0.97 (0.77–1.20)	0.90 (0.68–1.19)
⩾5	40	17 908	0.86 (0.61–1.21)	0.86 (0.61–1.21)	0.79 (0.51–1.22)
*P* for trend*			<0.05	<0.05	<0.05

Silhouette at age 20–25					
1	66	28 313	1.00 (reference)	1.00 (reference)	1.00 (reference)
2	418	133 410	1.33 (1.06–1.66)	1.35 (1.07–1.70)	1.26 (0.94–1.68)
3	293	111 187	1.12 (0.89–1.41)	1.14 (0.90–1.45)	1.13 (0.84–1.52)
4	97	35 457	1.14 (0.86–1.51)	1.13 (0.84–1.51)	1.11 (0.77–1.60)
⩾5	22	9314	0.99 (0.62–1.56)	1.01 (0.63–1.61)	0.97 (0.54–1.76)
*P* for trend*			NS	NS	NS

*Postmenopausal women*					
Silhouette at age 8					
1	1 357	349 402	1.00 (reference)	1.00 (reference)	1.00 (reference)
2	446	127 097	0.94 (0.85–1.05)	0.95 (0.85–1.05)	0.97 (0.85–1.10)
3	299	82 286	0.97 (0.86–1.10)	0.98 (0.86–1.11)	0.99 (0.85–1.15)
4	177	51 588	0.92 (0.79–1.08)	0.93 (0.79–1.09)	0.87 (0.72–1.07)
⩾5	49	16 973	0.77 (0.58–1.03)	0.76 (0.57–1.02)	0.69 (0.48–1.01)
*P* for trend*			<0.05	<0.05	<0.01

Silhouette around menarche					
1	598	148 043	1.00 (reference)	1.00 (reference)	1.00 (reference)
2	811	206 646	1.05 (0.96–1.16)	1.07 (0.97–1.18)	1.07 (0.95–1.21)
3	527	149 494	0.95 (0.85–1.06)	0.96 (0.86–1.08)	0.99 (0.86–1.14)
4	316	98 130	0.87 (0.77–1.00)	0.88 (0.77–1.01)	0.83 (0.70–0.98)
⩾5	120	35 545	0.91 (0.75–1.11)	0.91 (0.75–1.10)	0.84 (0.66–1.08)
*P* for trend*			<0.001	<0.001	<0.001

Silhouette at age 20–25					
1	296	72 926	1.00 (reference)	1.00 (reference)	1.00 (reference)
2	1 021	277 394	0.95 (0.84–1.06)	0.95 (0.84–1.07)	0.97 (0.84–1.13)
3	778	221 901	0.91 (0.80–1.02)	0.91 (0.80–1.04)	0.90 (0.75–1.03)
4	271	73 285	0.95 (0.81–1.11)	0.97 (0.82–1.13)	0.97 (0.79–1.17)
⩾5	80	21 625	0.95 (0.74–1.20)	0.94 (0.73–1.19)	0.93 (0.68–1.25)
*P* for trend*			NS	NS	NS

RR=relative risk; FFTP=first full-term pregnancy; NS=nonsignificant.

a Adjusted for menopause, age at menarche, age at FFTP, parity, marital status, number of years at school, height, alcohol consumption, familial history of breast cancer in first-degree relatives, personal history of benign breast disease, oral contraceptive use and physical activity.

b Additionally adjusted for BMI at baseline (1990), regularity of menstrual cycles when adult, interval between age at menarche and onset of regular cycling.

* Performed on the continuous variable ranked from 1 to 5+.

**Table 3:** Factors associated to a silhouette equal or greater than the 5th (logistic regression), at age 8 and around menarche. E3N study

	**Silhouette at age 8**			**Silhouette at menarche**		
**Variables**	**<5 (*n*)**	**⩾5 (*n*)**	**Adjusted OR^a^**	**<5 (*n*)**	**⩾5 (*n*)**	**Adjusted OR^a^**
*Age at menarche (years)*						
<12	16 752	692	1.00 (reference)	16 339	1389	1.00 (reference)
[12–13[	19 928	574	0.76 (0.68–0.85)	19 572	1331	0.85 (0.78–0.92)
[13–14[	20 129	439	0.60 (0.53–0.68)	19 908	1013	0.67 (0.61–0.73)
⩾14	22 774	453	0.56 (0.49–0.63)	22 595	955	0.57 (0.52–0.62)
*P* for trend			*P*<0.0001			*P*<0.0001

*Age at regular cycles* b *(years)*						
<12	30 622	921	1.00 (reference)	30 285	1900	1.00 (reference)
[12–13[	6244	193	0.88 (0.74–1.02)	6132	405	0.88 (0.79–1.00)
[13–14[	7794	212	0.92 (0.78–1.07)	7624	477	1.00 (0.90–1.11)
[14–15[	14 797	342	0.88 (0.78–1.01)	14 604	771	0.97 (0.89–1.06)
⩾15	20 115	490	1.04 (0.92–1.17)	19 769	1135	1.18 (1.10–1.28)
*P* for trend			*P*<0.01			*P*<0.01

*Interval between age at menarche and age at regular cycles*^b^ *(years)*						
=0	12 930	311	1.00 (reference)	12 787	630	1.00 (reference)
[0–1]	15 945	430	0.99 (0.88–1.12)	15 704	891	0.98 (0.90–1.06)
[1–2]	7942	189	0.88 (0.75–1.03)	7753	501	1.14 (1.03–1.26)
>2	16 237	478	1.14 (1.02–1.27)	15 918	1073	1.24 (1.15–1.34)
*P* for trend			*P*<0.01			*P*<0.01

*Regularity of menstrual cycling when adult*^b^ *(years)*						
Yes	69 430	1 836	1.00 (reference)	68 389	4028	1.00 (reference)
No	10 143	322	1.27 (1.12–1.44)	10 025	660	1.16 (1.07–1.27)

*BMI (kg/m* ^*2*^ *) at baseline (1990)*						
<20.4	22 050	279	1.00 (reference)	21 672	657	1.00 (reference)
[20.4–22.0]	21 631	448	1.43 (1.22–1.67)	21 112	967	1.35 (1.21–1.50)
[22.0–24.0]	21 703	609	1.72 (1.47–2.02)	20 925	1387	1.78 (1.59–1.98)
⩾24.0	20 699	801	1.73 (1.40–2.14)	19 875	1625	1.70 (1.46–1.97)
*P* for trend			*P*<0.0001			*P*<0.0001

OR=odds ratio.

a OR (95% CI) adjusted for menopause, age at menarche, age at FFTP, parity, marital status, number of years at school, height, BMI, alcohol consumption, familial history of breast cancer in first-degree relatives, personal history of benign breast disease, oral contraceptive use and physical activity.

b Age at menarche added as a confounder.

**Table 4:** RRs of breast cancer by body silhouette around menarche, according to menstrual characteristics and to BMI at inclusion E3N study, 1990–2000

**Variables**	**Cases (PY)**	**Multivariate RRs^a^**	**Cases (PY)**	**Multivariate RRs**	**Cases (PY)**	**Multivariate RRs**	**Cases (PY)**	**Multivariate RRs**
**Silhouette**	**Around 8 years old**				**Around menarche**			
	**Age at menarche <13 years**		**Age at menarche ⩾13 years**		**Age at menarche <13 years**		**Age at menarche ⩾13 years**	
1	819 (209 031)	1.00 (reference)	1017 (292 984)	1.00 (reference)	309 (77 554)	1.00 (reference)	458 (130 126)	1.00 (reference)
2	305 (93 580)	0.88 (0.77–1.01)	311 (101 096)	0.95 (0.83–1.07)	526 (131 807)	1.11 (0.98–1.27)	589 (172 116)	1.10 (0.98–1.23)
3	217 (65 286)	0.91 (0.78–1.06)	204 (61 049)	1.01 (0.87–1.18)	370 (112 425)	0.95 (0.82–1.10)	380 (114 027)	1.05 (0.92–1.20)
4	135 (42 654)	0.86 (0.72–1.03)	102 (35 201)	0.88 (0.72–1.07)	261 (80 332)	0.93 (0.79–1.09)	188 (69 606)	0.85 (0.72–1.01)
⩾5	40 (14 245)	0.75 (0.54–1.03)	29 (10 353)	0.83 (0.57–1.21)	81 (30 363)	0.76 (0.60–0.96)	78 (22 646)	1.07 (0.84–1.36)
*P* for trend		<0.01		=0.10		<0.001		<0.05

	**Interval ⩽1 year**		**Interval >1 year**		**Interval ⩽1 year**		**Interval >1 year**	
1	695 (185 744)	1.00 (reference)	537 (152 369)	1.00 (reference)	304 (78 266)	1.00 (reference)	221 (61 042)	1.00 (reference)
2	232 (71 241)	0.93 (0.80–1.07)	187 (59 523)	0.91 (0.81–1.02)	424 (113 320)	1.05 (0.91–1.21)	323 (90 287)	1.14 (1.02–1.28)
3	159 (45 640)	0.98 (0.82–1.16)	127 (39 729)	0.96 (0.83–1.09)	266 (81 983)	0.92 (0.79–1.08)	238 (70 799)	1.05 (0.93–1.19)
4	86 (26 931)	0.90 (0.72–1.13)	65 (25 006)	0.86 (0.72–1.02)	154 (51 725)	0.84 (0.69–1.01)	132 (48 614)	0.94 (0.82–1.08)
⩾5	24 (8 526)	0.75 (0.49–1.14)	18 (7 724)	0.80 (0.60–1.08)	62 (17 374)	1.00 (0.76–1.31)	39 (18 127)	0.84 (0.67–1.04)
*P* for trend		=0.07		<0.01		<0.05		<0.001

	**Irregular menstrual cycles in adulthood**		**Regular menstrual cycles in adulthood**		**Irregular menstrual cycles in adulthood**		**Regular menstrual cycles in adulthood**	
1	231 (65 299)	1.00 (reference)	1616 (439 696)	1.00 (reference)	114 (28 680)	1.00 (reference)	655 (180 126)	1.00 (reference)
2	67 (23 659)	0.88 (0.67–1.16)	552 (172 197)	0.92 (0.83–1.01)	124 (38 165)	0.96 (0.75–1.23)	1002 (267 688)	1.13 (1.03–1.24)
3	55 (16 504)	1.05 (0.78–1.40)	368 (110 590)	0.95 (0.85–1.06)	92 (28 397)	0.96 (0.73–1.26)	659 (199 407)	1.01 (0.91–1.12)
4	35 (10 166)	1.06 (0.74–1.52)	202 (68 130)	0.84 (0.73–0.98)	54 (19 037)	0.86 (0.62–1.18)	396 (131 809)	0.91 (0.80–1.02)
⩾5	10 (3 717)	0.86 (0.46–1.63)	60 (21 070)	0.78 (0.60–1.01)	21 (7 532)	0.82 (0.52–1.31)	139 (45 922)	0.90 (0.75–1.08)
*P* for trend		=0.50		<0.001		<0.05		<0.001

	**BMI <25 kg/m^2^**		**BMI ⩾25 kg/m^2^**		**BMI <25 kg/m^2^**		**BMI ⩾25 kg/m^2^**	
	**At baseline (1990)**		**At baseline (1990)**		**At baseline (1990)**		**At baseline (1990)**	
1	1553 (430 261)	1.00 (reference)	270 (69 036)	1.00 (reference)	669 (184 973)	1.00 (reference)	88 (21 522)	1.00 (reference)
2	520 (162 534)	0.93 (0.85–1.03)	95 (31 477)	0.84 (0.67–1.06)	940 (256 953)	1.11 (1.01–1.22)	177 (45 836)	1.12 (0.89–1.42)
3	329 (99 590)	0.95 (0.84–1.07)	92 (26 338)	1.02 (0.81–1.29)	619 (181 855)	1.04 (0.93–1.15)	130 (43 351)	0.98 (0.72–1.19)
4	176 (59 526)	0.85 (0.72–0.99)	57 (17 913)	0.93 (0.70–1.23)	342 (119 426)	0.86 (0.76–0.98)	102 (29 974)	1.05 (0.80–1.37)
⩾5	52 (17 789)	0.81 (0.61–1.07)	18 (6 769)	0.77 (0.47–1.26)	118 (39 707)	0.88 (0.72–1.07)	40 (13 193)	0.94 (0.65–1.35)
*P* for trend		<0.01		=0.20		<0.001		=0.22

RR=relative risk; BMI=body mass index; PY=person-years.

a djusted for menopause, age at menarche, age at FFTP, parity, marital status, number of years at school, height, alcohol consumption, familial history of breast cancer in first-degree relatives, personal history of benign breast disease, oral contraceptive use and physical activity.
